# Time Flows With the Changing of Events

**DOI:** 10.1111/nyas.70446

**Published:** 2026-10-29

**Authors:** Matthew Logie, Marianna Lamprou‐Kokolaki, Virginie van Wassenhove

**Affiliations:** ^1^ CEA, DRF/Joliot, NeuroSpin; INSERM; Cognitive Neuroimaging Unit Université Paris ‐ Saclay Gif/Yvette France

**Keywords:** cognition, cognitive psychology, psychology, salient, segmentation, time perception, unification

## Abstract

In recent years, investigations into the psychological experience of time have rapidly accelerated. The experience of time could depend on a ticking internal clock or rely on contextual changes within and between events. Cognitive theories of memory propose that the presence of event boundaries, as defined by unexpected changes, is crucial for the segmentation of sequenced content and ultimately, the formation of long‐term memories. Interestingly, the experience of change also influences the temporal distances between memories. Concurrent lines of research have developed similar theories on the experience of time, with salient contextual changes distorting temporal experience. Herein, we propose a unification of theories from the fields of time and memory. Events are creatively constructed and encoded into long‐term memory if sufficient changes occur. The subjective experience of time is also determined by an accumulation of changes creating temporal distances between memories. The encoding of memories provides temporal tags at multiple nested timescales, with no need to synchronize with the relative number of seconds that have passed, and duration estimates require a reconstruction of information between anchor points. Time does not exist without change, and experiencing change is fundamental for the formation of lasting memories.

## 1 Time and Memory

The human experience of time is driven by the tides of change, which appear to have an unchanging directionality; time flows from the past to the future and depends on memory for experienced changes. The brain segments present experiences and compresses and stores them in memory as episodes that define the past. Segmenting the continuous flow of experiential changes provides the anchor points for the encoding of memories, while the rate of change may define the distance between memories and, quite possibly, the experience of time itself. Herein, we argue that temporal experiences depend on the rate of change and the construction, compression, and encoding of information in memory. Specifically, our first claim is that parsing information is not parsing time: chunking a stream of information does not entail representing the interval spanned, which is a constraint that neither the discussed event segmentation theory (EST) [1] nor contextual‐change accounts [2, 3] state explicitly. While they assume that the event carries its own duration, we argue it does not [4, 5]. Second, we propose that time tags, and not durations per se, are the long‐term storage format for the *when* of memory: durations are distances computed between tags at retrieval. The computation of distances between tags has been argued for animal learning [6]; applying it to human event memory and deriving retrospective estimation from it is something the timing literature has not fully addressed [5, 7, 8]. Third, compression rate is the quantity that predicts underestimation. Information accumulated between boundaries sets a compression rate, so that the greater the compression of information, the greater the underestimation of duration. Focusing on the compression and imperfect reconstruction of information changes the functional relation between time and information that is distinct from the restatement of “more changes mean longer” typically found in the literature. Last, we suggest that attention acts as a sampling rate: attending to time increases temporal resolution. Altering the sampling of information alters compression, subsequent reconstruction, and the computation of temporal distances. An altering of the sampling of information between events provides a different prediction than the regular ticks of an internal clock of scalar expectancy theory (SET), since it should affect the reconstruction latency, not just the estimate [9, 10, 11].

 “It is utterly beyond our power to measure the changes of things by time. Quite the contrary, time is an abstraction, at which we arrive by means of the change of things” [12]

Theories of time experience within psychology have traditionally been based on the assumption of an internal clock [13, 14], wherein a pacemaker provides a stable temporal metric for the passage of time by counting ticks in an accumulator from which a duration can be derived. This model assumes a highly stable and stationary oscillatory mechanism to provide a consistent temporal metric, a requirement that is idealized and somewhat unrealistic in neural systems [7, 15, 16]. The notion of a centralized, dedicated timekeeping system is also not parsimonious, and neuroscientific implementations have not provided satisfactory accounts for multiple aspects of timing. Instead, alternative models, such as those proposing a decay rate of event memory across multiple time scales [17, 18, 19, 20] or state‐dependent networks in which the complexity of the network gives rise to timing [21, 22], seem to offer more biologically plausible implementations.

Given that time is not a physical quantity that can be directly perceived and measured, some models have adopted a more cognitive, information‐processing approach proposing that perceived duration is constructed based on perceptual content [23]. Behavioral evidence supports this view: prospective duration estimates increase with the number of perceptual changes occurring during an interval. More changes lead to longer perceived durations [24, 25, 26] and conversely, sensory adaptation to a constant rate of change results in a compression of perceived duration of subsequent stimuli [27, 28]. Perceptual time and duration in particular can be described in terms of the number of items [23] or changes in the stimulus [29]. However, in most studies, the definition of contextual change is highly limited and refers to simple, low‐level sensory features, such as pixel changes in a video stream [26]. While this sensory‐based, feedforward approach to temporal cognition has been appealing due to its similarities with magnitude perception, it often equates temporal experiences with sensory inputs, conflating information processing with timing. Indeed, it has long been suggested that the construction of duration depends not only on the number of items but also on how they are organized [3], and very few models in timing have incorporated this idea (but see [30]).

Herein, we would like to consider changes not as sensory features but as realistic atoms of computation for the estimation of time in real life, that is, as events and episodes. A crucial implication of this consideration is that an event has an extent in time [31]. Although life is experienced as a continuous flow of events [1], the contents of human memory are composed of temporally discrete episodes. These discrete events or episodes provide a key feature of human cognition that provides organizational principles for the structuration of events in the brain as opposed to sensory events. Additionally, we challenge an important limitation of traditional models that instantiate duration as the primary code for temporal processing, as they fail to account for temporal order. This is essential because event and interval timing have been dissociated as relying on a phase and an interval code, respectively [8]. Below, we will discuss how memory models can contribute to our understanding of how the brain represents time, not as the duration of events but through temporal tagging of events, which can be fully reconciled with a phase or an event‐coding scheme. In that sense, duration arises as a distance computation between two events in memory [6] and does not constitute the primary format by which time is encoded.

We will first introduce EST and accompanying notions of event boundaries and context drift. Then, we explain how these approaches can provide alternative views to the forward coding of temporal information as well as the recollection of the timing of events. We then report how the internal clock from SET cannot fully account for the estimation of durations and the *when* of memory. Last, we propose a pathway toward unifying theoretical accounts of time and memory.

## 2 Event Segmentation: Hierarchical Time

The ability to hold and manipulate information within the mind at the present moment, such as the words within the current sentence, can be defined as working memory [32]. In contrast, episodic memory involves the encoding, storage, and retrieval of long‐term memories of autobiographical events [33]. Event‐based memory, as described by EST [34], is thought to be an account of the cognitive processes involved in creating events and seeks to explain the interactions that occur between working and episodic memory. EST proposes that the formation of episodic memories depends upon segmenting the continuous flow of information encountered throughout life. An event, a segment of experience with a specific spatiotemporal context, is represented in working memory as an event model, allowing predictions to be formed about what will happen next. When what occurs does not match the prediction, a prediction error is elicited, which acts as a trigger for encoding the contents of working memory into long‐term episodic memory [35]. Thus, EST provides a way to define long‐term memory whereby prediction errors act as event boundaries that demarcate the beginning and end of an event maintained within working memory, but also an episode encoded in long‐term memory. Crucially, information constructed in working memory can be lost without being subsequently available for reconstruction [36]. The absence of sufficient contextual change leads to a loss of information as clear events are unavailable for compression, encoding, and subsequent reconstruction from long‐term memory.

If an event boundary has been experienced, then memory tasks are necessarily testing information that has already been encoded into long‐term memory. Participants show poorer memory performance after experiencing boundaries that can be defined in terms of spatial or temporal gaps [37] because information has been cleared from working memory, requiring an (imperfect) reconstruction of the representation of the event [38]. Studies of walking through doorways support this interpretation [39], as do studies of memory for objects in films [40]. However, there is evidence that event boundaries can be defined solely in terms of contextual change without any prediction error occurring [41, 42]. For example, enhancing the predictability of boundaries by removing doorways suggests that improving predictability does not prevent the segmentation of events from occurring [43]. Long‐term memory performance and memory for temporal duration and order may thus be influenced by contextual shifts [44].

Interestingly, the process of event segmentation operates across multiple hierarchical timescales [45], from fine‐grained events at the sensory level to more high‐level, coarse‐grained segmentation [46, 47]. It is, therefore, unsurprising that segmentation at all timescales also appears to shape one's experience of duration. At the finest‐grained sensory level, unexpected or salient stimulus changes can affect duration estimates: studies demonstrate distorted temporal judgments for unexpected stimuli [48, 49, 50], including visual [51], auditory [52], and multisensory perception [53]. Even low‐level perceptual dynamics, such as object motion or saccadic eye movements, modulate time perception [25, 54, 55], likely by introducing segmentation points. At a mid‐level scale, physical transitions, like turning corners, walking through doorways, or entering new environments, act as segmentation cues that affect both memory and duration estimates [43, 56, 57]. Similarly, changes in musical structure that include nontemporal contextual changes mark the start and end of multiple bands of temporal sequences, such as unexpected changes in pitch (frequency) or loudness (amplitude) [58]. At higher scales, broader shifts in context, such as being at work, at home, or on holiday, serve as anchors for event segmentation and alter our sense of time [18, 59, 60].

Notably, segmentation is not limited to external sensory or contextual cues. Emotional shifts can also act as powerful markers of change, sometimes exerting stronger grouping effects than spatial or temporal cues alone [61, 62, 63]. In a study of segmentation, participants were presented with sequences of images alongside musical passages. Event boundaries were provided with changes in the music, such as valence or arousal. After 24 h, surprise memory tests were performed with a recognition test of old and new images and placing an image on a timeline. Participants were more accurate in placing an image on a timeline if the image had occurred at an event boundary, perceptual changes in music, valence, and arousal [61]. The study supports the view that events may be defined in terms of changes in context, and changes of context can be provided in a variety of ways.

Thus, contextual changes are a pervasive property of life experiences that help the segmentation of information at different hierarchical scales. The structuring of information into units (events, episodes) influences one's perception of time.

## 3 Event‐Based Timing

One current major limitation of models of time is that they rely on sensory perception that uses an impoverished definition of event, which has not yet incorporated the event sequences of memory as described in EST [1]. Herein, we suggest that a similar event‐based account of memory is also an event‐based account of time perception [6, 64]. Accordingly, the subjective experience of time depends upon the computed temporal distances between events in memory without a dedicated duration storage.

Experiences of duration have long been proposed to depend upon the number and complexity of contextual changes that occur in both external stimuli and internal mnemonic activities [2, 3, 65]. Support for this view can be found in a study of task switching [66]. The subjective experience of time passing was examined by either providing a journal article to read, watching an entertaining sitcom, or switching between reading the article and watching the sitcom. Participants experienced the most time passing when watching the sitcom and the least time passing when reading the article. The experience of time passing for switching between the two occupied a middle ground between reading the article and watching the sitcom. One interpretation of the result could be due to the experience of contextual changes that accelerate contextual drift and create temporal distance between memories. From this perspective, the greater number of contextual changes experienced when watching the sitcom are the cause of the increased estimation of time passing. Notably, the results of task switching studies will depend on individual differences in the activities that individuals find engaging [67]. Additionally, the subjective experience of time can also be influenced by inhibition and mental updating [68]. Task switching, inhibition, and updating may be more generally described in terms of contextual shifts, which update the mental representations held within working memory, an interpretation that we will return to.

In the study of time perception, clear distinctions have been made between prospective and retrospective timing [69, 70]. Retrospective judgments depend upon the number of contextual changes that occur in a given interval, whereas prospective judgments also depend upon the allocation of attentional resources directed at keeping time. It is also noteworthy that cognitive systems crave information load [71, 72]. When information load is nonoptimal, false memories can be fabricated in order to fill the gaps [73, 74]. Based on studies of the construction of false memories, increased prospective duration estimates may be due to internal mnemonic activities during unfilled gaps rather than increased attention toward counting pulses. Accordingly, both prospective and retrospective judgments of timing are due to the number of contextual changes that occur. Contextual changes can be provided by both internal mnemonic constructions, such as structured thought and mind wandering, and perceptually salient stimuli. Differences between prospective and retrospective judgments may thus depend upon both internal and external contextual changes.

Evidence in favor of time and memory as a record of change can be found in studies based on behavioral work, computational modeling, and neuroimaging [75]. When comparing duration estimates of human participants and an image classifying an artificial neural network, both the humans and the neural network were found to produce similarly distorted duration estimates [26]. For the neural network, durations were recorded based on the dynamics of image classification determined by salient changes in sensory inputs, even though the network was not trained to mimic human duration estimates. The distorted duration estimates of both the network and the humans included perceiving short durations as longer and long durations as shorter than they actually were, which is consistent with Vierordt's law or the central tendency effects of Bayesian models [76]. Additionally, while matching the number of seconds of presentation, scenes that contained a lot of visual information were experienced as lasting longer than scenes containing less visual information. The duration of time was shown to depend on the dynamics of perceptual classification and the accumulation of salient changes as predicted by earlier models [2, 3, 23, 24, 29, 65].

A later study combined a behavioral and computational modeling approach to link salient perceptual change to prediction errors and working memory to episodic memory [18]. In the model, episodic memories were encoded if a sensory event did not match a prediction and successive events created a nested structure. The study demonstrated that both prospective and retrospective duration estimates depended on the number of predictable and unpredictable changes experienced. Prospective judgments were driven by the number of changes encoded, while retrospective judgments were driven by the number of changes recalled. Interestingly, for the computational model, duration estimates depended upon the rate of changes per second within a particular band of nested salient changes (i.e., perception or working memory or episodic memory). Similar duration estimates were found for human participants, supporting the interpretation of duration estimates and memory performance depending upon the most relevant layer of a nested record of change.

Altogether, these findings demonstrate that segmenting and hierarchically organizing events in memory across timescales is central to both memory and time perception. Accordingly, recent work continues to question the continuity in time [77], challenging the intuitive notion of continuous experience, in favor of discrete models of consciousness. From a perceptual awareness standpoint, this question is recurrent [5, 78] because it raises the question of how one moment may be distinguished from the next in the experiential flow of time. Indeed, evidence suggesting that experiential time relies on the endogenous structuration of (discrete) events is strongly counterintuitive, as it clashes with the phenomenology of a continuous flow of time. How then, in the face of EST, is a seemingly continuous experiential flow or chronology maintained?

## 4 Context Drift and Ordinality

A fundamental basis for establishing the chronology of memories is the representation of context. Contextual representations drift at a certain rate, and the similarity of context determines temporal distances between items to be remembered. A theoretical account of this can be found in the temporal context model [79]. According to the temporal context model, contextual similarity provides a grouping effect in memory. Experiencing highly salient changes shifts the contextual representation, creating temporal gaps between groups of items which share a contextual overlap [80]. The rate of drift fluctuates based on the experience of change, and increasing the rate of contextual drift increases temporal distance even when no additional seconds have passed [57]. Additionally, experiencing event boundaries has been shown to provide a reset to the start of event context [81]. The segmentation of context both increases the number of items that can be recalled and improves memory for temporal order. Temporal clustering also improves memory performance for items that are close in time due to a shared context; items appearing further apart in time are separated due to a decreased contextual overlap. Furthermore, memory retrieval has been linked with the reinstatement of contextual detail [82], suggesting that such details can cue the retrieval of subsequent events in the stored memory sequence. As an example, when tasked with recalling short word lists, participants will tend to recall the words in a similar order to the order of initial presentation [83].

Overall, these studies point to a central role of temporal context in memory organization and performance. Supporting this idea, research on reading and narrative comprehension shows that readers perceive temporal shifts (e.g., “an hour later,” “the next day”) as event boundaries that help them structure the narrative in time [84]. Similarly, in studies using sequences of images with category‐based boundaries (e.g., switching between faces and objects), event segmentation impacts temporal memory performance [85, 86]. Crucially, temporal memory relies on context reactivation, which, in the case of an image sequence, is the reactivation of the representation of surrounding items [85]. Boundaries seem to disrupt temporal context by strengthening within‐event associations while weakening across‐event associations [85, 86, 87, 88]. It is also noteworthy that recent work describes the importance of neural drifts in the temporal structure of experiences [89, 90], although a direct causal link between neural drifts and the experience of time has not yet been established [91].

The presentation of contextual changes provided by spatial shifts has also been shown to influence the experience of time [92]. In this study, participants were presented with filmed scenes and asked to make estimates of how long each scene lasted. The scenes either displayed characters moving through doorways (spatial shifts) or characters moving without passing through doorways. Time estimates made at retrieval were found to be longer for scenes that contained spatial shifts than for scenes that did not contain spatial shifts. One interpretation of these data is that the spatial shift experienced when passing through doorways decreases contextual overlap, increases temporal distance between events, and acts as a temporal marker for memory. Similarly, in the study of spatial navigation [56], turns in a maze increased the recollected duration of the event.

The effects of contextual change have also been explored within virtual environments [74]. Participants were asked to remember paintings presented in different locations within a virtual reality environment. Memory performance for sequentially probed paintings depended upon the temporal distance between paintings without discrete changes provided by passing through doorways. The closer the temporal distance, the better the memory performance. Previous studies have also shown that contextual changes can disrupt memory for temporal order. While memory performance may be influenced by temporal clustering without the need for definite boundaries between clusters [43]. Boundaries defined by doorways, spatial gaps, and context shifts have been interpreted as driving segmentation by provoking a prediction error as described by EST [1, 40, 93, 94]. A recent virtual reality study highlighted how the quality (salience or distinctiveness) of spatial contexts define events and provide anchor points for the *what* of future memories [95]. Contextual shifts between distinct virtual contexts also provide strong anchor points for the *when* of memory, which influence the temporal distances between items in memory. Segmenting groups of images in distinct contexts provides structure for the temporal compression of memory, with images placed closer together on a timeline than originally presented. The compression occurred at nested scales both within each virtual room (event) and across the entire sequence [96, 97]. Not only were the items compressed, but items occurring earlier in an event were placed closer together than items occurring later within an event. The results provide evidence for the compression, encoding, decompression, and reconstruction of information within events driving the subjective experience of episodic time.

Note that while virtual reality can provide a particularly effective approach to studying segmented contextual change with tight controls on spatial and temporal gaps, emotional boundaries have also been shown to influence the temporal compression of memory. Compression depends on the sampling rate of information within events, which in turn determines subsequent temporal errors. A recent study showed that negative emotions drive an increase in the sampling rate, which reduces the compression of information and increases the subjective experience of time [98]. Taken to the extreme, negative emotions in depressive states may be defined as a dysfunction of segmentation. Recent electroencephalography work on rumination in depressive states provides further evidence for emotions influencing the sampling of information and the segmentation of events [99, 100, 101]. Beyond spatial and temporal gaps and changes of emotions, the memorability of the images themselves can drive segmentation effects. Memorability is a property of an image that determines how likely an image is to be remembered. Presenting a sequence of low memorability images followed by a sequence of high memorability images also provides a sufficient contextual change that alters the temporal distances between items in memory [102]. The compression and reconstruction of information determine the temporal distances between items in memory.

Hence, contextual changes (boundaries) provided by spatial shifts improve memory performance and provide anchor points for the temporal compression of information. Estimations of time also expand based on the sampling rate of information within events, which increases with negative emotions. Taken together, the result suggests that time depends on the sampling of information driving changes in how much contextual drift has occurred between boundaries. The concepts of space and time along with the concepts of valence and arousal of emotions may all contribute to the rate of change determining the compression of information and the subjective experience of time [103].

## 5 Time and Nested Event Sequences

Rather than being encoded as informational content dependent on the number of items [32] or increasing noise [104], the contents of working memory may be encoded in terms of complexity of change [105]. Recent work has argued that episodic memory may be composed of multiple phases of temporally limited memory buffers [59, 106]. The episodic buffer [107] has recently been recharacterized as an awareness buffer [108] and could be further defined as a layer of episodic memory with the lightest compression and the shortest temporal limit [109]. The buffer may hold information in a lightly compressed state, maintaining accessibility for a fully decompressed processing of information within dedicated subcomponents of working memory. Single events are constructed within working memory. Experiencing sufficient contextual change drives the compression and encoding of an event into a phase of memory that can store sequences of compressed events, limited by the number of contextual shifts that can be stored [110]. Both the subjective experience of time and the formation of memories can be defined in terms of a record of change within nested sequences. With events encoded within successively longer‐lasting phases of memory [111]. Duration estimates and memory performance would then depend upon the rate of memory compression and the most relevant layer of the nested record of change [112].

Taken together, fluctuating rates of change may govern the subjective experience of time and the construction of memories by increasing temporal distance between mnemonic packets within nested temporal sequences. Time perception depends on the inherent complexity of capturing packets of information within cognitive systems, consistent with state‐dependent networks [22], as was also suggested by behavioral studies and computational modeling [75]. While memories may pass through phases of temporally limited episodic buffers [111], we must also account for how the temporal limits of memory are defined. One possibility, based on the study of entropy and Bayesian surprise [113, 114], is that memory processes involve tracking an accumulation of change; current contextual representations are compressed and encoded into longer‐lasting memory when the accumulation reaches a threshold. From this perspective, humans generate time with the processing of information and the encoding and reconstruction of a new experience.

## 6 Boundaries, Context Drift, and the Experience of Time

An established account for the memory of durations and interval timing comes from the development of interval‐based internal clock models [14, 115]. SET [116] provides an influential account of an internal clock in which time perception depends upon an oscillatory pacemaker mechanism that generates pulses. The number of pulses generated within a given interval is read by an accumulator. The pacemaker, regulated by physiological factors such as arousal, starts when an event begins. Attention regulates the latency at which pulses are read by the accumulator and held within working memory. The current pulse count can be stored in a reference memory for comparison with subsequent counts. The system could also record the timing of multiple simultaneous events [117]. The initial formation of internal clock models could account for a linear experience of time. Studies employing psychophysical methods demonstrated that the subjective experience of time was not linear and could vary based on salient sensory stimuli [4, 118]. To account for variations in subjective time experience, attention was given the role of keeping track of pacemaker pulses, such that when attending to alternative stimuli, fewer pulses would be recorded. For example, if attention is distracted by sensory stimuli, pulses from the pacemaker may be missed, which provides an explanation for subjective time experience as the absence of attention to time yields to an underestimation of duration. While the findings on the underestimation of duration are robust, there are alternative theoretical explanations than that of a dedicated pacemaker, which we address in the following section.

One of the traditional theories on the subjective experiences of time comes from the concept of contextual change. The more contextual change experienced, the more time passes [2, 23]. One potential drawback of the internal clock account is the need for an observer that is dedicated to the clock [119]. This account leads to a similar issue faced within working memory research on executive functioning. Positing the existence of a central executive opened the possibility of an infinite hierarchy of managers described as internal homunculi [120] or decision demons that scream when a particular stimulus is perceived [121]. Much like approaches to explain executive functions and the complexity of cognition [122, 123], timing mechanisms may instead arise from the interaction of multiple networks rather than being dependent upon attention or a hierarchy of dedicated managers.

In favor of the interacting network view, subjective time experience can be found in the development of state‐dependent networks [21, 22]. The inherent complexity of interacting neural networks and the recurrent processes they produce may give rise to the subjective experience of time without the need for a dedicated internal clock mechanism. Indeed, online timing could be well accounted for by intrinsic synaptic scaling and temporal basis functions, such as those described in the cerebellum for subsecond timing [124]. However, they do not provide a mechanistic account of how durations are stored. Timing models strongly assume that time is coded as a duration in long‐term memory, thus that a magnitude (duration or interval) of time is available, namely, that a discrete storage of moments or scalar magnitudes is available. Such an assumption is congruent with observations of central tendencies and priors in duration estimation, given a learned set of durations in the lab that can be contextually modulated [125] or even stronger societal priors observed in single‐shot retrospective time estimations in real‐life experiments [126, 127]. The storage of durations is also congruent with recent work suggesting that sequences of durations can be held in working memory [128, 129].

The experience of time passing has also been shown to depend on the intensity of changes in ongoing visual perception [130]. In that study, participants viewed a starfield (provided by colorful dots traveling on a screen) within which the velocity and density of stars along with the duration of the viewing experience could be manipulated. Both the velocity and the density of the stars altered the experience of the passage of time. A higher rate of change provided by increasing the velocity or density of the starfield accelerated time such that more time was experienced. However, manipulating the number of seconds that participants viewed the starfield did not affect the experienced passage of time. Similarly, duration and passage of time are affected by the rate of events. Increasing the number of events lengthens duration estimates, and the rate of change accelerates the passage of time. Duration estimates required longer reaction times than passage of time judgments, suggesting that duration estimates may require a reconstruction of the number of changes that occurred [131]. In a previous study, paying attention to time lengthens duration estimates, whereas not attending to time shortens estimates. Additionally, increasing the load placed on working memory leads to reductions in duration estimates [132]. From a contextual drift perspective, the quantity of information accumulation within an event alters the passage of time by increasing contextual dissimilarity, which determines temporal distances and the rate of compression of information, which leads to an underestimation of duration. See Table 1 and Figure 1.

**TABLE 1:** Nine explananda and three explanans.

***Phenomenon***	***Internal clock (SET, pacemaker−accumulator)***	***Contextual change/drift***	***Time tags + compression*** ***(present account)***
*Stored format of temporal information*	*Duration is a scalar magnitude in reference memory* [14]	*Duration is inferred from contextual dissimilarity at retrieval* [23, 29, 79]	*Duration is not stored. Events carry time tags, durations are computed as a distance between event tags* [6, 133, 134]
*Memory for temporal order*	*Not derivable as pulse counts are not ordered* [8]	*Derivable from similarity of contextual representations, and ordinality is a byproduct* [57, 81, 85, 86, 88]	*Order is primary, based on distance from event boundary (tag) duration is secondary, based on a difference of reconstructed distances* [6, 34, 133]
*Equivalent number of changes; distinct temporal structure*	*No effect (same pulse count)* [3] *but see* [30]	*No effect, or unspecified because change count is the variable* [96, 105]	*Estimates diverge: temporal structure sets the compression rate, so that structured intervals are more compressed and more underestimated* [109, 133, 135]
*Retrieval latency: duration versus passage‐of‐time*	*Both are read from the accumulator with no principled asymmetry between the two*	*Unspecified*	*Duration requires reconstruction across tags and should be systematically slower; passage judgments read the rate of change directly* [131]
*Retrieval latency for an item within an interval*	*Independent of boundary structure*	*Weakly dependent via contextual overlap*	*Scales with the number of intervening boundaries and distance to nearest boundary* [142]
*Attention to time*	*Fewer (more) missed pulses yield longer (shorter) estimates* [14, 115, 116, 132]	*Finer encoding of changes yield longer estimates*	*Higher sampling rate yields lower compression thus longer estimates. It also predicts changed reconstruction latency* [133]
*Working memory load*	*Lost pulses predict shorter estimates* [132]	*Fewer changes encoded yield shorter estimates*	*Quantity of information between boundaries defines compression rate and the difference in distances* [109, 110, 112, 132, 133]
*Segmentation without prediction error*	*Not addressed*	*Compatible: any contextual shift will drift context* [42, 43, 44]	*Compatible and required: tagging is triggered by contextual shift, not by error* [41, 133]
*Within‐ versus across‐event distortion*	*Uniform scaling*	*Uniform drift within events with an acceleration or resetting of contextual representations at boundaries* [57, 81]	*Simultaneous contraction within events and dilation across them. Nestedness across scales* [109, 110, 112, 133]

The subjective experience of time can be explained as a result of task engagement or in terms of curiosity for seeking out new experiences, which can provide us with prediction errors and improve our future predictions. In this case, the best forms of media entertainment are designed to provide prediction errors, surprise, and provoke, all of which drive engagement. However, a scientist with expertise and passion for a subject will be more engaged in reading the papers related to their field of interest. We form predictions, then seek experiences to test those predictions in nested loops throughout life. We have a certain agency in driving our experience of time through the tasks that we choose to engage with. Given a choice, we choose to engage with tasks that require us to update our memories and form better predictions; being denied a choice can lead to dysfunctions of temporal experiences by becoming either under‐ or overwhelmed with unpredictable change. Engagement, or attention to a task, is a concept that could more formally be described as a sampling of information. The experience of time is fundamentally dependent on the quantity of information, the structure of events, and the records of change within memory. Some studies are starting to tackle this complex notion by adapting lab experiments to real‐life scenarios [67].

**FIGURE 1:**
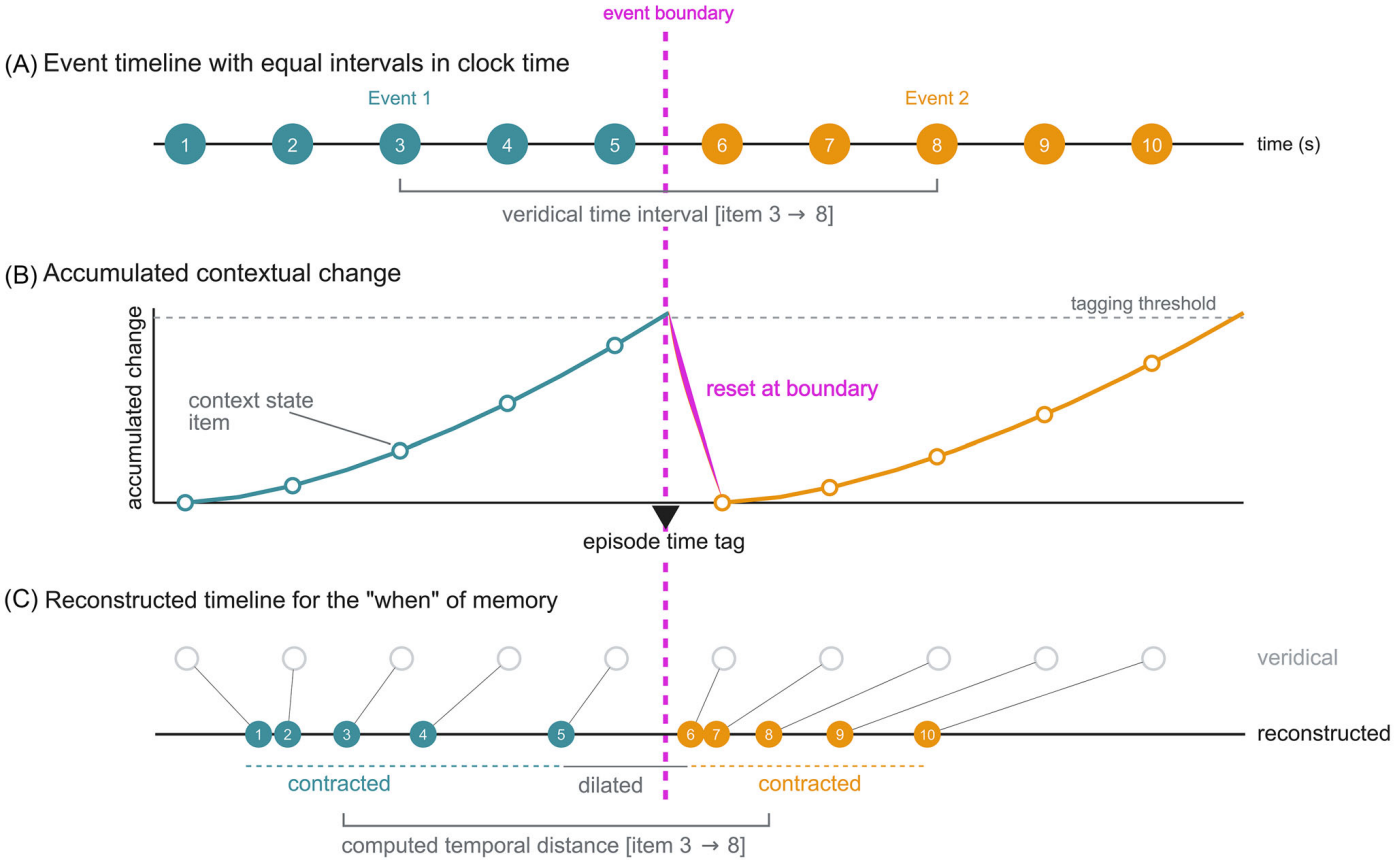
Context drift and rate of change.  (A) Segmented sequence of to‐be‐remembered items defined by equal intervals. (B) Rate of change builds up and resets at an event boundary, establishing the encoding of a compressed event and creating a temporal tag for subsequent retrieval. (C) Temporal distances depend on how much change occurred between images, with larger distances between items 3‐4‐5 than items 1‐2‐3. The reconstructed timeline reflects the coexistence of drift, compression, and imperfect reconstruction appearing as contractions and dilations in the *when* of memory. Note that repetition of a similar event (such as the same types of items with a change in background color) can result in event 2 displaying a higher rate of compression than event 1, and that the size of contractions and dilations may depend on the size of prediction error between events and differing rates of compression at the level that is tested. This explanation is also consistent with recent findings [133].

The sampling and compression of information between event boundaries determines the rate of compression, which can alter temporal distances. Remembering *when* depends on the decompression and imperfect reconstruction of information, which may also alter temporal distances. Recent behavioral work has established how the nested structure of events drives temporal distances between items in episodic memory [133]. However, questions remain as to how the compression of information at encoding and the decompression and reconstruction at retrieval each contribute to the temporal distances between memories and the subjective experience of time. Specifically, looking for markers with EEG during both encoding and retrieval of segmented stimuli may provide the clearest answers with evidence of temporal compression and the establishment of temporal tags in memory [136]. We would expect participants to flexibly adapt their strategies for either forward or backward scanning of memory based on the task. Differences in temporal distances depend on the quantity of information within nested event structures and how the to‐be‐remembered information aligns with narrative schema [137].

The construction of memory and the experience of time may depend on temporal integration windows delineated by context shifts, which could be described as packets within oscillatory cycles [138] or presemantic windows [134, 139]. The retrieval of memories involves the creative reconstruction of past events [140, 141], and a study of segmentation has suggested that the basis for reconstruction involves skipping across encoded event boundaries. When asked to retrieve a target item from a particular movie segment, participants’ reaction times to identify an item depended upon the number of boundaries and the distance between the item and the previous boundary [142]. It is important to note that although participants were not required to provide judgments of the passage of time or duration estimates, the time to retrieve the memory for a target item depended upon the number of boundaries and the distance between the target item and the closest boundary.

While segmentation effects can be provided by external contextual shifts, critically, shifts can also be internally generated. Task switching has been shown to influence the experience of time [66] and has also been shown to create boundaries that impair memory for temporal order [143]. Experiencing either externally or internally generated change provides segmentation effects in memory; the construction of episodic memories and the subjective experience of duration may be described in terms of the segmentation of events or a fluctuating contextual drift. However, rather than distinguishing between segmentation and contextual drift, the two theoretical accounts may both be correct. An alternative view is that durations are not stored but events are temporally marked, allowing to compute their distances in time [6, 144]. Crucially, temporal order cannot be explained by internal clock models, whereas a temporal marker account also provides an order with memory for events reaching into the past and imaginatively constructed events reaching into the future.

In summary, in memory research, segmentation parses streams of information into meaningful bits called events and episodes that are hierarchically nested. However, it is essential to dissociate the parsing of information from the parsing of time. If information is segmented or chunked, this does not warrant that the time interval associated with the beginning and the end of the information segment is itself represented [145, 146]. By representation, we mean “a symbolic read‐write memory that carries information extracted from experience forward in time in a computationally accessible form” [6]. Herein, we suggest that salient moments like event boundaries used in memory parsers, which imply contextual change, may be temporally tagged in memory. This time‐tagging operation may be a more parsimonious solution than duration storage for real‐time temporal coding of singular life events. One implication is that long‐term storage of temporal information is not duration but time tags [6, 146]. Context shifts may provide salient markers of time passing by virtue of being privileged moments for coding temporal information. Temporal distances in the event model can be determined by how much contextual change occurs; the compression of information provides an explanation for how retrospective duration tends to be underestimated. The quantity of information experienced between boundaries determines a compression rate. Time estimates require the computation of information; the greater the compression rate, the more time will be underestimated. Attending to time increases the sampling rate (temporal resolution) of information processing (seeing the trees rather than the forest), which alters the compression rate and the subsequent decompression and reconstruction of information when making duration estimates.

## 7 Conclusion

The current article highlights the potential development of unified theories of time and memory. Daily personal experiences are composed of creative constructions of events that are encoded into long‐term memory if sufficient changes occur; similarly, changes influence temporal distance and the subjective experience of time. Studies of time and memory may benefit from converging on the study of fluctuating context drift. The *rate of drift* may be influenced by one's experience of change, which determines the rate of compression and the contractions and dilations of time in the imperfect reconstruction of memories on the timelines of our lives. Time flows with the changing of events.

## Author Contributions

**Matthew Logie**: conceptualization, writing – original draft, writing – review and editing, visualization. **Marianna Lamprou‐Kokolaki**: conceptualization, writing – review and editing. **Virginie van Wassenhove**: conceptualization, writing – review and editing, visualization, supervision, project administration, funding acquisition.

## Conflicts of Interest

All authors declare no conflicts of interest

## Data Availability

Data sharing is not applicable to this article as no datasets were generated or analyzed during the current study.
